# Sodium sulfite hepta­hydrate and its relation to sodium carbonate hepta­hydrate

**DOI:** 10.1107/S2053229620004404

**Published:** 2020-04-20

**Authors:** Matthias Weil, Kurt Mereiter

**Affiliations:** a Institute for Chemical Technologies and Analytics, Division of Structural Chemistry, TU Wien, Getreidemarkt 9/164-SC, A-1060 Vienna, Austria

**Keywords:** sulfite, O—H⋯S hydrogen bonding, crystal structure, crystal chemistry, hepta­hydrate, structural similarity

## Abstract

The monoclinic crystal structure of Na_2_SO_3_(H_2_O)_7_ is characterized by an alternating stacking of (100) cationic sodium–water layers and anionic sulfite layers along [100]. The cationic layers are made up from two types of [Na(H_2_O)_6_] octa­hedra that form linear ^1^_∞_[Na(H_2_O)_4/2_(H_2_O)_2/1_] chains linked by dimeric [Na(H_2_O)_2/2_(H_2_O)_4/1_]_2_ units on both sides of the chains. The isolated trigonal–pyramidal sulfite anions are connected to the cationic layers through an intricate network of O—H⋯O hydrogen bonds, together with a remarkable O—H⋯S hydrogen bond, with an O⋯S donor–acceptor distance of 3.2582 (6) Å, which is about 0.05 Å shorter than the average for O—H⋯S hydrogen bonds in thio­salt hydrates and organic sulfur com­pounds of the type *Y*—S—*Z* (*Y*/*Z* = C, N, O or S). Structural relationships between monoclinic Na_2_SO_3_(H_2_O)_7_ and ortho­rhom­bic Na_2_CO_3_(H_2_O)_7_ are discussed in detail.

## Abstract 2

The crystal structure of Na_2_SO_3_(H_2_O)_7_ shows close structural similarities with Na_2_CO_3_(H_2_O)_7_, though the two hepta­hydrates belong to different crystal systems (monoclinic and ortho­rhom­bic, respectively) and contain anions with different shapes.

## Introduction   

Sodium sulfite is used extensively in industrial processes, for example, as an anti­oxidant and preservative in food industries (E number for food additives E221), as a corrosion inhibitor in aqueous media, as a bleaching agent, as a solubilizing agent for cellulose, straw and wood in the pulp and paper industry, or as an additive in dying processes. In the USA alone, the production of sodium sulfite reached 150 000 tons in 2002 (Weil *et al.*, 2007 ▸). Solid sodium sulfite is stable in its anhydrous form and as the hepta­hydrate. Despite its use at industrial scales, structural details are known only for anhydrous Na_2_SO_3_ that crystallizes with two formula units in the trigonal system in the space group *P* 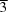 (Larsson & Kierkegaard, 1969 ▸). Bond lengths and near-neighbour distances of sodium sulfite in aqueous solution have been calculated by *ab initio* quantum mechanical charge field mol­ecular dynamics (QMCF MD) studies and determined experimentally by large-angle X-ray scattering (LAXS) by Eklund *et al.* (2012 ▸). For crystalline Na_2_SO_3_(H_2_O)_7_, lattice parameters and the space group (*P*2_1_/*n*) have previously been determined from Weissenberg photographs without providing further structural details, except for a close metrical resemblance with ortho­rhom­bic Na_2_CO_3_(H_2_O)_7_ (Dunsmore & Speakman, 1963 ▸). To obtain a more detailed picture of the relationship between the hepta­hydrates of Na_2_SO_3_ and Na_2_CO_3_, we grew single crystals of Na_2_SO_3_(H_2_O)_7_ and determined its crystal structure. Indeed, the two hepta­hydrates show not only a close metrical relationship (Table 1 ▸), but also structural similarities, though they belong to different crystal systems and contain differently shaped divalent anions, *viz*. trigonal–pyramidal SO_3_^2−^ and trigonal–planar CO_3_^2−^.

## Experimental   

### Crystallization   

Colourless prismatic crystals of Na_2_SO_3_(H_2_O)_7_ were grown by recrystallization of a commercial anhydrous sample (Merck, p.A. grade) from an aqueous solution at room tem­per­ature by slow evaporation over the course of several days. In order to remove adherent mother liquor, the crystals were placed on filter paper and subsequently immersed in Paratone oil. The crystal under investigation was cleaved from a larger specimen.

### Crystallography and refinement   

Crystal data, data collection and structure refinement details are summarized in Table 2 ▸. The crystal structure of Na_2_SO_3_(H_2_O)_7_ was originally solved and refined in the space group *P*12_1_/*n*1 (No. 14), with lattice parameters *a* = 11.8576 (8), *b* = 7.2197 (5), *c* = 12.6965 (9) Å and β = 106.7938 (17)° at 100 K (full crystal data in the setting *P*12_1_/*n*1 are available in CIF format as supporting information). The values for the lattice parameters at 100 K are in good agreement with the previous study, with values of *a* = 11.922, *b* = 7.260, *c* = 12.765 Å and β = 107.22° obtained at room temperature from Weissenberg film data (note that *a* and *c* are inter­changed in the original description; Dunsmore & Speakman, 1963 ▸). For a better com­parison with the reported crystal structure of β-Na_2_CO_3_(H_2_O)_7_ (Betzel *et al.*, 1982 ▸), the nonconventional setting *C*112_1_/*a* was chosen for the final structural description of Na_2_SO_3_(H_2_O)_7_, using the matrix (101, 10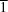, 010) for transformation of the primitive cell to the *C*-centred cell with *c* as the unique axis; moreover, the atomic coordinates and the origin of the unit cell were chosen to ensure a similar packing of structural building blocks in the two unit cells of Na_2_SO_3_(H_2_O)_7_ and β-Na_2_CO_3_(H_2_O)_7_. All H atoms present in the crystal structure of Na_2_SO_3_(H_2_O)_7_ were located in a difference Fourier map and were refined freely.

## Results and discussion   

### Crystal structure   

**Figure 1:**
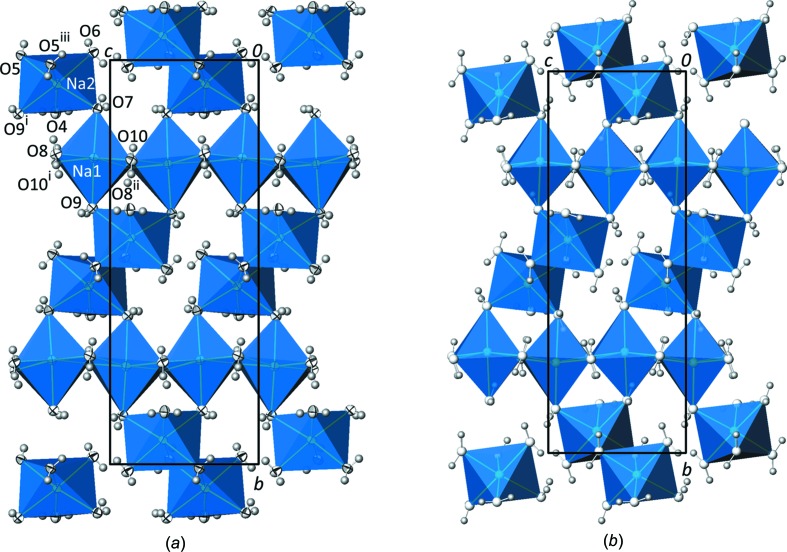
(*a*) View along [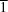
00] onto the cationic water–sodium (100) layer in the crystal structure of Na_2_SO_3_(H_2_O)_7_ made up from edge- and corner-sharing [Na(H_2_O)_6_] octa­hedra (turquoise). Anisotropic displacement ellipsoids are drawn at the 90% probability level and H atoms are shown as grey spheres of arbitrary radii. Symmetry codes refer to Table 3 ▸. (*b*) The same type of layer in the crystal structure of Na_2_CO_3_(H_2_O)_7_, with atoms as spheres of arbitrary radii.

In the crystal structure of Na_2_SO_3_(H_2_O)_7_, all atoms (2 Na, 1 S, 10 O and 14 H) are located on general sites. The two sodium cations are surrounded by six water mol­ecules, defining a distorted octa­hedral coordination polyhedron in each case. The Na—O distances range from 2.3690 (6) to 2.4952 (6) Å (Table 3 ▸), with mean values of 2.42 (4) Å for Na1 and 2.43 (6) Å for Na2, in fairly good agreement with the mean value for Na^[6]^—O of 2.44 (11) Å calculated for 5520 individual bonds (Gagné & Hawthorne, 2016 ▸). The bond valence sums (Brown, 2002 ▸) for the sodium cations, as calculated with parameters provided by Brese & O’Keeffe (1991 ▸), are 1.15 valence units (v.u.) for Na1 and 1.13 v.u. for Na2, and thus in the expected range for monovalent Na^+^. The O—Na—O angles deviate clearly from ideal values, with values for *trans* O atoms in the range 172.149 (16)–176.42 (2)° for Na1 and 165.81 (2)–174.23 (2)° for Na2, and for *cis* O atoms in the range 81.464 (19)–101.74 (2)° for Na1 and 81.51 (2)–103.23 (2)° for Na2. The two types of [Na(H_2_O)_6_] octa­hedra show a different linkage pattern. Octa­hedra centred by Na1 share common edges (O8/O10 and O8^ii^/O10^i^; see Table 3 ▸ for symmetry codes) to form infinite linear ^1^_∞_[Na1(H_2_O)_4/2_(H_2_O)_2/1_] chains running parallel to [001], whereas octa­hedra centred by Na2 make up dimeric [Na2(H_2_O)_2/2_(H_2_O)_4/1_]_2_ units by sharing an edge (O5 and O5^iii^). In both cases, the corresponding Na—O bonds to the shared O atoms at the edges are the shortest in the respective octa­hedron. The dimeric units connect adjacent chains by sharing the terminal water mol­ecules (O9 and O7) on both sides of the chains (corner-sharing links). This way, the sodium–water octa­hedra are assembled by edge- and corner-sharing into an infinite layer extending parallel to (100) (Fig. 1 ▸ *a*).

**Figure 2:**
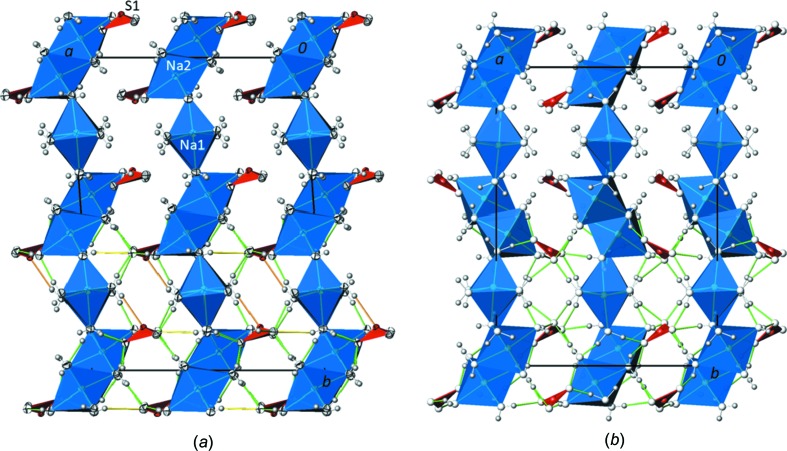
(*a*) The crystal structure of Na_2_SO_3_(H_2_O)_7_ in a projection along [001], showing the layered character with cationic water–sodium layers at *x* ≃ 0, 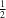 alternating with sulfite layers (red polyhedra) at *x* ≃ 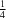, 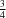. (*b*) The crystal structure of Na_2_CO_3_(H_2_O)_7_ in a projection along [00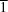], showing the same type of layer stacking but a different orientation of the dimeric groups and adjacent carbonate anions at *y* ≃ 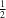. For clarity, hydrogen bonds are displayed only in the lower half of the figures, with moderate O—H⋯O hydrogen bonds shown as green lines, weak O—H⋯O bonds as yellow lines and O—H⋯S hydrogen bonds as orange lines.

The sulfite anion has the characteristic trigonal–pyramidal configuration, with the S^IV^ atom occupying the pyramidal position. Atom S1 is 0.5912 (4) Å above the basal plane formed by atoms O1, O2 and O3. The S—O bond lengths are in a narrow range 1.5224 (5)–1.5338 (5) Å [mean 1.527 (6) Å], just like the O—S—O angles [105.85 (3)–106.07 (3)°; mean 105.93 (16)°]. Again, these values are in good agreement with the grand mean S^IV^—O bond length of 1.529 (15) Å calculated for 90 bonds and with the O—S^IV^—O angles in the range ∼99–107° with a mean value of ∼104° (Gagné & Hawthorne, 2018 ▸). The bond valence sum for atom S1 is 4.12 v.u., using the parameters of Brese & O’Keeffe (1991 ▸) for calculation. The sulfite anions are isolated from the sodium–water layer, lying alternatingly on both sides outside of an individual layer. In this way, cationic sodium–water layers at *x* ≃ 0, 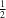 are sandwiched by sulfite layers at *x* ≃ 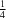, 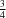 and stacked along [100], with the sulfite anions situated approximately at the height in *y* where the [Na2O_2/2_O_4/1_]_2_ dimers are linked to the ^1^_∞_[Na1(H_2_O)_4/2_(H_2_O)_2/1_] chains (Fig. 2 ▸ *a*).

**Figure 3:**
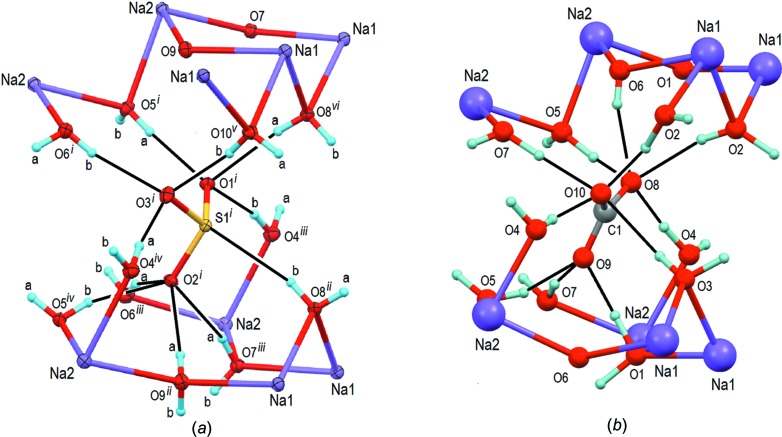
Com­parison of the hydrogen bonding to the anion in (*a*) Na_2_SO_3_(H_2_O)_7_, with displacement ellipsoids drawn at the 50% probability level, and (*b*) Na_2_CO_3_(H_2_O)_7_, with atoms as arbitrary spheres; hydrogen bonds are shown as thin solid lines. Atoms O1 and O3 in the sulfite structure accept three hydrogen bonds each, whereas O8 and O10 in the carbonate structure accept four each. Likewise, O2 in the sulfite structure accepts four hydrogen bonds, whereas the corresponding O9 atom in the carbonate accepts three. Note that the arrangement of the hydrogen-bonded water mol­ecules around SO_3_^2−^ is approximately mirror-symmetric (*e.g.* O5^i^ and O6^i^), whereas it is less symmetric for the carbonate. Symmetry codes for atoms in Na_2_SO_3_(H_2_O)_7_ involved in hydrogen bonding with the SO_3_^2–^ anion are: (i) *x*, *y*, *z*; (ii) *x* + 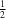, *y* − 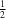, *z*; (iii) −*x* + 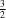, −*y*, *z* + 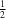; (iv) −*x* + 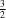, −*y*, *z* − 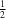; (v) −*x* + 1, −*y*, −*z* + 2; (vi) −*x* + 1, −*y*, −*z* + 3.

The seven independent water mol­ecules possess approximately tetra­hedral coordination arrangements (including hydrogen bonds), except for O9, and five of them each bridge two sodium cations (O5, O7, O8, O9 and O10), whereas two are each bonded to only one sodium cation (O4 and O6). An intricate network of O—H⋯O hydrogen bonds between the water mol­ecules and the sulfite O atoms link the anionic layers to adjacent cationic layers, thus establishing a three-dimensional hydrogen-bonded network structure (Fig. 2 ▸ *a*). Based on the donor–acceptor distances between 2.7204 (7) and 2.9110 (8) Å (Table 4 ▸), the hydrogen-bonding strength is moderate according to the classification of Jeffrey (1997 ▸). Most of these hydrogen bonds are donated to sulfite atoms O1, O2 and O3 (Fig. 3 ▸ *a*). Thereby, atom O1 is the acceptor of three, O2 of four and O3 of three hydrogen bonds. It is worth noting that the S—O bond lengths reflect this situation nicely, with S1—O2 = 1.5338 (5) Å being about 0.01 Å longer than the remaining two. The O9 water mol­ecule, bonded to Na1, Na2 and *via* H9*A* to O2, lacks a clearcut hydrogen bond for its second H atom (H9*B*), which points to H6*B* of the O6—H6*B*⋯O3 hydrogen bond [H9*B*(*x*, *y* − 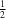, *z* + 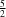)⋯H6*B* = 2.49 Å], while distances from O9(*x*, *y* − 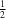, *z* + 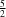) to O6 and O3 exceed 3.3 Å.

In addition to the inter­actions between water mol­ecules and sulfite O atoms, there are two hydrogen bonds between water mol­ecules only (O7⋯O6^vi^ and O10⋯O4^ii^; symmetry codes refer to Table 4 ▸), and, as a pecularity, an O—H⋯S hydrogen bond between O8 and S1^vii^. In general, S⋯H inter­actions involving divalent S atoms are considered as weak hydrogen bonds (Allen *et al.*, 1997 ▸). The H⋯S hydrogen-bonding strength becomes even weaker for H⋯SO_3_ contacts because the S atom is positively polarized in an SO_3_^2−^ anion with partial double-bond character for the S—O bonds (Nyberg & Larsson, 1973 ▸). The hydrogen-bond acceptor ability of divalent sulfur was evaluated some time ago from 1811 substructures of mostly organic com­pounds, *i.e. Y*—S—*Z* systems (*Y*/*Z* = C, N, O or S) as acceptor groups retrieved from the Cambridge Structural Database, giving a mean inter­molecular >S⋯H distance of 2.67 (5) Å for O—H donors and a mean S⋯O distance of 3.39 (4) Å (Allen *et al.*, 1997 ▸; Groom *et al.*, 2016 ▸). In com­parison, the first ever reported crystal structure determination of an inorganic com­pound with an O—H⋯S hydrogen bond and a clear location of the H atoms, *viz*. BaS_2_O_3_(H_2_O) from neutron single-crystal diffraction data (Manojlović-Muir, 1969 ▸), revealed a considerably shorter S⋯H distance of 2.367 (4) Å and a likewise shorter S⋯O distance of 3.298 (4) Å. The O—H⋯S angle in BaS_2_O_3_·H_2_O was determined as 163 (3)°. Corresponding values of the O—H⋯S hydrogen bond in the crystal structure of Na_2_SO_3_(H_2_O)_7_ are somewhat larger at 2.455 (14) Å for H8*B*⋯S1^vii^ (X-ray data), slightly shorter at 3.2582 (6) Å for O8⋯S1^vii^ and similar at 164.5 (13)° for the O8—H8*B*⋯S1^vii^ angle. A com­parable O⋯S distance of 3.326 Å was found as the mean value for 86 hydrogen-bonding inter­actions between water mol­ecules and S atoms in a variety of thio­salt hydrates, such as Schlippes salt, Na_3_SbS_4_(H_2_O)_9_ (Mikenda *et al.*, 1989 ▸). A literature search indicated that the O—H⋯S hydrogen bond in the title com­pound appears to be unprecedented thus far among hydrated sulfites. This suggests that in sulfite hydrates, O—H⋯O hydrogen bonding is clearly preferred over O—H⋯S hydrogen bonding and that a certain structural motif is needed to induce O—H⋯S hydrogen bonding like in the title com­pound. Invoking the results of an electron deformation density study of MgSO_3_(H_2_O)_6_ (Bats *et al.*, 1986 ▸), the coordination capability of the sulfite S atom *via* its electron lone-pair lobe at the apex of the SO_3_ pyramid is not unexpected, but this capability seems to be weak in the context of hydrogen bonding because otherwise more examples with features com­parable to the title com­pound would have been encountered already. As soon as covalent bonding comes into play, the coordination capability of the sulfite S atom is well documented by transition-metal com­plexes like K_2_[Pd(SO_3_)_2_]·H_2_O (Messer *et al.*, 1979 ▸) or K_2_[Hg(SO_3_)_2_]·2.25H_2_O (Weil *et al.*, 2010 ▸), with metal–sulfur bonds, or by hydrogen sulfites like CsHSO_3_ (Johansson *et al.*, 1980 ▸) or K_5_(HSO_3_)(S_2_O_5_) (Magnusson *et al.*, 1983 ▸) that contain HSO_3_^−^ anions with hydrogen covalently bonded to sulfur.

The numerical values of the atomic distances for crystalline Na_2_SO_3_(H_2_O)_7_ (Tables 3 ▸ and 4 ▸) are in good agreement with those of aqueous Na_2_SO_3_ solutions determined from LAXS studies, with S—O = 1.53 Å for the sulfite group and Na—O = 2.41 Å for the sodium—water distances (Eklund *et al.*, 2012 ▸). In the latter study, the S⋯O_water_ distance in solution was determined as 3.68 Å, which is considerably longer than in the solid state, giving further evidence for a weak but existing O—H⋯S hydrogen bond in the crystalline material.

### Com­parison with Na_2_CO_3_(H_2_O)_7_   

The close structural relationship between monoclinic Na_2_SO_3_(H_2_O)_7_ and ortho­rhom­bic Na_2_CO_3_(H_2_O)_7_ (Table 1 ▸) becomes evident from the similar arrangement of the principal building units in the crystal structures. The same type of cationic sodium–water layers made up from edge- and corner-sharing [Na(H_2_O)_6_] octa­hedra [mean Na—O distance = 2.43 (4) Å and O—Na—O angles = 81–102 and 164–180°; Fig. 1 ▸ *b*] is present in the carbonate, likewise situated at *x* ≃ 0, 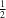 in the unit cell (Fig. 2 ▸ *b*). The carbonate groups do not show pyramidalization (Zemann, 1981 ▸) and occupy the same space as the sulfite groups between adjacent layers close to the [Na(H_2_O)_2/2_(H_2_O)_4/1_]_2_ dimers.

The main difference between the two structures is related to the orientation of the [Na(H_2_O)_2/2_(H_2_O)_4/1_]_2_ dimers in the layers. Whereas in the sulfite structure, the dimers at *y* ≃ 0 and 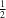 in one layer and also the accom­panying anions close to them have the same orientation relative to (100), in the carbonate structure, the orientation of every second dimer (at *y* ≃ 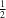) and the accom­panying anions in a layer is reversed due to the presence of the *c*-glide plane (Fig. 2 ▸).

The hydrogen-bonding schemes in the two hepta­hydrates are similar (Fig. 3 ▸). In the carbonate structure, the anions are likewise hydrogen bonded to water mol­ecules through medium–strong hydrogen bonds [O⋯O = 2.690 (5)–3.060 (4) Å, with an additional weak inter­action of 3.223 (5) Å]. In analogy, two water–water O—H⋯O inter­actions with donor–acceptor distances of 2.827 (5) and 2.766 (5) Å are also observed. However, in contrast to the central sulfite S atom with its free electron lone pair, the central C atom of the carbonate anion cannot act as a hydrogen-bond acceptor, and thus this inter­action is missing in the carbonate structure.

### Com­parison with related com­pounds   

Crystal structures with sulfite groups anchored exclusively by hydrogen bonds are at present restricted to the title com­pound Na_2_SO_3_(H_2_O)_7_, to NH_4_SO_3_(H_2_O) (Battelle & Trueblood, 1965 ▸; Durand *et al.*, 1977 ▸) and to MgSO_3_(H_2_O)_6_ (Andersen & Lindqvist, 1984 ▸; Bats *et al.*, 1986 ▸). In MgSO_3_(H_2_O)_6_, which is built up from [Mg(H_2_O)_6_] octa­hedra and isolated SO_3_ pyramids within a lattice of the space group type *R*3, and with Mg and S atoms both located on threefold rotation axes, there are two independent water mol­ecules that donate, apart from one water–water hydrogen bond, three water–O_sulfite_ hydrogen bonds to each sulfite O atom, com­parable to O1 and O3 in Na_2_SO_3_(H_2_O)_7_, but with shorter O⋯O distances [2.687 (3), 2.701 (3) and 2.726 (3) Å] than in the latter. An electron deformation density study of MgSO_3_(H_2_O)_6_ (Bats *et al.*, 1986 ▸) proved the presence of an extended lone-pair lobe at the apex of the SO_3_ pyramid, but neither MgSO_3_(H_2_O)_6_ nor NH_4_SO_3_(H_2_O) contain O—H⋯S or N—H⋯S hydrogen bonds.

A further com­parison with other hydrated sodium com­pounds com­prised of related oxo anions shows no close structural relationship to the title hepta­hydrate. For example, Na_2_SO_4_(H_2_O)_7_ (Oswald *et al.*, 2008 ▸) (*I*4_1_/*amd*, *Z* = 4) has a com­pletely different arrangement of the principal building units. Its crystal structure is com­prised of [Na(H_2_O)]_6_ octa­hedra concatenated by edge- and corner-sharing into a three-dimensional network with isolated tetra­hedral sulfate anions hydrogen bonded to the chains. Also, for sodium com­pounds with analogous trigonal–pyramidal oxoanions and the same charge, *i.e.* *X*O_3_^2−^, with *X* = Se and Te, no phases related structurally or com­positionally to Na_2_SO_3_(H_2_O)_7_ are known. For Na_2_SeO_3_, the anhydrous form (*P*2_1_/*c*, *Z* = 4) is made up from [NaO_6_] octa­hedra and trigonal–pyramidal SeO_3_^2−^ anions (Wickleder, 2002 ▸), and is isotypic with Na_2_TeO_3_ (Masse *et al.*, 1980 ▸). Hydrated forms are known only for the penta­hydrates Na_2_SeO_3_(H_2_O)_5_ (Mereiter, 2013 ▸) and Na_2_TeO_3_(H_2_O)_5_ (Philippot *et al.*, 1979 ▸) that are, surprisingly, not isotypic (*Pbcm*, with *Z* = 8, and *C*2/*c*, with *Z* = 8, respectively). These structures are based on two- or three-dimensional assemblies of [NaO_5_] polyhedra (Se) and [NaO_6_] octa­hedra (Se and Te), to which SeO_3_/TeO_3_ groups are bonded *via* two (Se) or one (Te) O atom. The [NaO_6_] octa­hedra in these two salts share common faces and edges but no vertices. As pointed out by Philippot *et al.* (1979 ▸) for Na_2_TeO_3_(H_2_O)_5_ and confirmed also for Na_2_SeO_3_(H_2_O)_5_ (Mereiter, 2013 ▸), the electron lone pair of Se and Te in these structures shows no attracting inter­actions with neighbouring H atoms. This might be one reason why hydrates of Na_2_SeO_3_ and Na_2_TeO_3_ do not crystallize in the Na_2_SO_3_(H_2_O)_7_ structure and *vice versa*. 

## Supplementary Material

Crystal structure: contains datablock(s) I, global. DOI: 10.1107/S2053229620004404/ep3004sup1.cif

Structure factors: contains datablock(s) I. DOI: 10.1107/S2053229620004404/ep3004Isup2.hkl

CIF with full numerical data (setting P121/n1). DOI: 10.1107/S2053229620004404/ep3004sup3.txt

Supporting information file. DOI: 10.1107/S2053229620004404/ep3004Isup4.cml

CCDC reference: 1993827

## Figures and Tables

**Table 1:** Com­parison of lattice parameters (Å, °) for Na_2_SO_3_(H_2_O)_7_ (this work) and Na_2_CO_3_(H_2_O)_7_ (Betzel *et al.*, 1982 ▸)

	Na_2_SO_3_(H_2_O)_7_	Na_2_CO_3_(H_2_O)_7_
*a*	14.6563 (8)	14.492 (7)
*b*	19.7180 (9)	19.490 (5)
*c*	7.2197 (5)	7.017 (3)
α	90	90
β	90	90
γ	94.0997 (17)	90
*V* (Å^3^)	2081.1 (2)	1981.95
*T* (K)	100	RT
Space group	*C*112_1_/*a*	*Pbca*

**Table 2:** Experimental details

Crystal data	
Chemical formula	Na_2_SO_3_(H_2_O)_7_
*M*_r_	252.15
Crystal system, space group	Monoclinic, *C*112_1_/*a*
Temperature (K)	100
*a*, *b*, *c* (Å)	14.6563 (8), 19.7180 (9), 7.2197 (5)
γ (°)	94.0997 (17)
*V* (Å^3^)	2081.1 (2)
*Z*	8
Radiation type	Mo *K*α
μ (mm^−1^)	0.42
Crystal size (mm)	0.15 × 0.13 × 0.12

Data collection	
Diffractometer	Bruker APEXII CCD
Absorption correction	Multi-scan (*SADABS*; Krause *et al.*, 2015 ▸)
*T*_min_, *T*_max_	0.675, 0.747
No. of measured, independent and observed [*I* > 2σ(*I*)] reflections	16909, 4845, 4222
*R*_int_	0.021
(sin θ/λ)_max_ (Å^−1^)	0.827

Refinement	
*R*[*F* ^2^ > 2σ(*F* ^2^)], *wR*(*F* ^2^), *S*	0.023, 0.063, 1.06
No. of reflections	4845
No. of parameters	174
H-atom treatment	All H-atom parameters refined
Δρ_max_, Δρ_min_ (e Å^−3^)	0.89, −0.33

Com­puter programs: *APEX2* (Bruker, 2014 ▸), *SAINT* (Bruker, 2014 ▸), *SHELXT* (Sheldrick, 2015*a* ▸), *SHELXL2018* (Sheldrick, 2015*b* ▸), *ATOMS* (Dowty, 2006 ▸), *Mercury* (Macrae *et al.*, 2020 ▸) and *publCIF* (Westrip, 2010 ▸).

**Table 3:** Selected geometric parameters (Å, °)

Na1—O10	2.3690 (6)	Na2—O4	2.3939 (6)
Na1—O8	2.3785 (6)	Na2—O7	2.4093 (6)
Na1—O10^i^	2.4199 (6)	Na2—O6	2.4928 (6)
Na1—O7	2.4199 (6)	Na2—O9^i^	2.4952 (6)
Na1—O8^ii^	2.4436 (6)	S1—O3	1.5224 (5)
Na1—O9	2.4599 (6)	S1—O1	1.5234 (5)
Na2—O5^iii^	2.3787 (6)	S1—O2	1.5338 (5)
Na2—O5	2.3805 (6)		
			
O3—S1—O1	105.85 (3)	O1—S1—O2	105.87 (3)
O3—S1—O2	106.07 (3)		

Symmetry codes: (i) 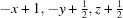; (ii) 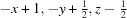; (iii) 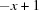, 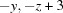.

**Table 4:** Hydrogen-bond geometry (Å, °)

*D*—H⋯*A*	*D*—H	H⋯*A*	*D*⋯*A*	*D*—H⋯*A*
O4—H4*A*⋯O3^iv^	0.800 (15)	2.031 (16)	2.8216 (8)	169.7 (15)
O4—H4*B*⋯O1^v^	0.821 (16)	1.983 (16)	2.7904 (7)	167.8 (15)
O5—H5*A*⋯O1	0.804 (13)	1.947 (13)	2.7503 (7)	175.9 (13)
O5—H5*B*⋯O2^iv^	0.798 (15)	1.994 (15)	2.7694 (7)	163.9 (15)
O6—H6*A*⋯O2^v^	0.777 (14)	2.072 (14)	2.8206 (7)	161.8 (14)
O6—H6*B*⋯O3	0.774 (15)	1.962 (15)	2.7204 (7)	166.5 (15)
O7—H7*A*⋯O2^v^	0.810 (13)	1.976 (14)	2.7761 (7)	169.3 (13)
O7—H7*B*⋯O6^vi^	0.773 (14)	2.171 (14)	2.9110 (8)	160.7 (15)
O8—H8*A*⋯O1^iii^	0.792 (15)	2.009 (15)	2.7900 (7)	168.9 (13)
O8—H8*B*⋯S1^vii^	0.825 (14)	2.455 (14)	3.2582 (6)	164.5 (13)
O9—H9*A*⋯O2^vii^	0.807 (14)	2.106 (14)	2.9096 (7)	174.0 (14)
O10—H10*A*⋯O4^ii^	0.839 (15)	1.962 (15)	2.7908 (8)	169.5 (14)
O10—H10*B*⋯O3^vi^	0.762 (14)	2.069 (14)	2.8210 (7)	169.1 (14)

Symmetry codes: (ii) 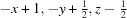; (iii) 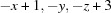; (iv) 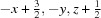; (v) 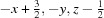; (vi) 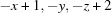; (vii) 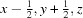.
